# Is there any relationship between mutation in CPS1 Gene and pregnancy loss?

**DOI:** 10.18502/ijrm.v17i5.4604

**Published:** 2019-06-26

**Authors:** Mehrdad Talebi, Mohammad Yahya Vahidi Mehrjardi, Kambiz Kalhor, Mohammadreza Dehghani

**Affiliations:** ^1^Department of Medical Genetics, Shahid Sadoughi University of Medical Sciences, Yazd, Iran.; ^2^Medical Genetics Research Center, Shahid Sadoughi University of Medical Sciences, Yazd, Iran.; ^3^Department of Biological Science, Faculty of Science, University of Kordestan, Sanandaj, Iran.; ^4^Reproductive and Genetic Unit, Yazd Research and Clinical Center for Infertility, Shahid Sadoughi University of Medical Sciences, Yazd, Iran.

**Keywords:** CPS1 deficiency, Hyperammonemia, Urea cycle disorder, Whole exome sequencing.

## Abstract

**Background:**

Carbamoyl phosphate synthetase 1 (CPS1) is a liver-specific enzyme with the lowest enzymatic rate, which determines the overall rate of the other reactions in the pathway that converts ammonia to carbamoyl phosphate in the first step of the urea cycle. Carbamoyl phosphate synthetase 1 deficiency (CPS1D), which usually presents as lethal hyperammonemia, is a rare autosomal recessive hereditary disease.

**Case:**

We report a case of a two-day-old female neonate with lethal hyperammonemia. The newborn infant was presented with hyperammonemia (34.7 μg/ml; reference range 1.1–1.9). In Plasma amino acid analysis, there was a significant elevated levels of alanine (3,004 μmol/L; reference range, 236–410 μmol/L), glutamine (2,256 μmol/L; reference range, 20–107 μmol/L), asparagine (126 μmol/L; reference range, 30–69 μmol/L), glutamic acid (356 μmol/L; reference range, 14–192 μmol/L), aspartic acid (123 μmol/L; reference range, 0–24 μmol/L), and lysine (342 μmol/L; reference range, 114–269 μmol/L). We cannot diagnose the urea cycle disorder (UCD) CPS1D properly only based on the quantity of biochemical intermediary metabolites to exclude other UCDs with similar symptoms. Following next generation sequencing determined one homozygous mutation in CPS1 gene and also this mutation was determined in her parents. The identified mutation was c.2758G > C; p.Asp920His, in the 23 exon of CPS1. This novel homozygous mutation had not been reported previously.

**Conclusion:**

We applied whole exome sequencing successfully to diagnose the patient with CPS1D in a clinical setting. This result supports the clinical applicability of whole exome sequencing for cost-effective molecular diagnosis of UCDs.

## 1. Introduction

The urea cycle is the primary function of the liver that is highly conserved in all mammalian species, this process Converting toxic ammonium into the less toxic urea(1, 2). There are six enzymes in urea cycle, including Carbamoyl phosphate synthetase 1 (CPS1), N-acetylglutamate synthase (NAGS), ornithine carbamoyl transferase (OTC), arginosuccinate synthetase, arginosuccinate lyase, and arginase. High ammonium concentration in the blood caused by the deficiency in any of these enzymes can have harmful effects as serious as anorexia, central nervous system dysfunction, brain damage, lethargy, coma, and even death (3, 4). CPS1 converts ammonia to carbamoyl phosphate in the first step of the urea cycle (5). The incidence of Carbamoyl phosphate synthetase 1 deficiency (CPS1D) was reported to be 1/62000 in the United States (5), 1/539000 in Finland (6), and 1/800000 in Japan (7). CPS1D has been classified into two different clinical phenotypes based on the activity level of the CPS1 enzyme, clinical manifestations, and the age of onset. These two groups included Neonatal onset and late onset and the first one that manifests clinically within the first few days of life is Neonatal onset CPS1D. The affected infant was characterized by refusal to feed, hypotonia, lethargy, convulsions, hypothermia, vomiting, coma, and even death (8).

By contrast, there are less severe clinical manifestations associated with later onset CPS1D. CPS1, which is located on 2q35, is a large gene, spanning over 120 kb, consisting of over 38 coding exons, which is about 4500 coding nucleotides (9).

As described in the Leiden Open Variation Database (LOVD, http://www.LOVD.nl/CPS1) and the Human Gene Mutation Database (HGMD, http://www.hgmd.org/), there are More than 240 CPS1 pathogenic variations that are reported to be enormously distributed among the coding exons in CPS1 pathogenic variants. Just about 10% of the identified pathogenic variants take place in unrelated cases, predominantly affecting CpG dinucleotides, further complicating diagnosis due to the “private” nature of such pathogenic variants (10).

Previously, the relationship between mutation in the CPS1 and homocysteine has been reported in women, and homocysteine is found frequently in women with RPL. Inborn metabolic disorders have been considered as a harmful factor for mother, embryo, and the pregnancy (11, 12). However, an increasing number of evidence propose that fetal inborn metabolic disorders can be associated with serious medical problems including fetal cardiomyopathy, structural defects of brain, isolated ascites or hydrops fetalis, congenital malformation, and fetal death (13).

This article aims to highlight the consequences of inborn metabolic disease of the fetus on the pregnancy outcome and to suggest a metabolic work up for cases with clinical problems relating to pregnancy because this family has experienced three pregnancy losses.

## 2. Case Presentation

We report a two-day-old female neonate with symptoms including poor feeding, flatulence, and lethargy that started a day after her birth. Her weight at birth was 3010 gr, and the patient had no facial dysmorphism or other phenotypic abnormalities at birth. At admission, primary physiological neurologic reflex was observed. Her blood tests showed hyperammonemia (34.7 ug/ml; reference range, 1.1–1.9). Plasma amino acid analysis revealed markedly elevated levels of alanine (3,004 μmol/L; reference range, 236-410 μmol/L), glutamine (2,256 μmol/L; reference range, 20–107 μmol/L), asparagine (126 μmol/L; reference range, 30–69 μmol/L), glutamic acid (356 μmol/L; reference range, 14–192 μmol/L), aspartic acid (123 μmol/L; reference range, 0–24 μmol/L) and lysine (342 μmol/L; reference range, 114–269 μmol/L). Collectively, these results suggested a urea cycle disorder (UCD).

### Genetic test

**Figure 1:**
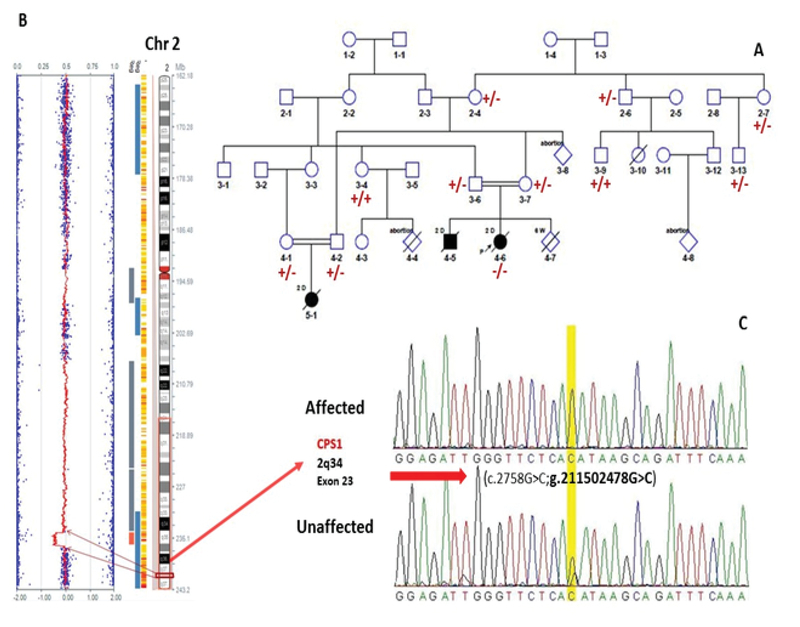
(A) Pedigree of family with individuals affected by Carbamoyl phosphate synthetase 1 deficiency (CPS1D). Segregation of mutation in the family with the mutation allele is shown by –, and wild type is shown by +. (B) SNP array profile of the case; 2 Mb delition region on chr 2q37. (C) Sanger sequence of the CPS1 gene revealed c.2758G > C mutation.

After genetic counseling and collection of the parents' informed consents cytogenetic analysis was performed using peripheral blood of the patient and the parent, based on G-banding and standard phytohemagglutinin-stimulated lymphocyte. Cytogenetic analysis revealed a normal karyotype. We performed Single Nucleotide Polymorphism (SNP) array using Illumina Human CytoSNP-12 V2.1 bead-chip array. Subsequent analyses using Genome Studio V2010.2 were performed to analyze the generated data. We defined almost 2 Mb deletion region on chr 2q37. The result from analyses revealed that the genes presented in deleted sites are not correspondent to patient symptoms. Afterward, we performed a whole exome genome sequencing based on next generation sequencing (NGS; Illumina platform). For aligning sequences, we used GATK, BWA, and ANNOVAR software to identify variants and annotating, respectively. After the filtration of all identified variants, we found a novel missense c. 2758G> C mutation in exon 23 of CPS1 at amino acid position 920 (p. Asp920His). At the end, we used the Sanger sequencing to confirm that the mutation was in the patient (homozygous). The mutation was checked in her parents and other family members too (Figure 1).

## 3. Discussion

CPS1 converts ammonia to carbamoyl phosphate in the first step of the urea cycle and CPS1 gene, which is located on 2q35, is a large gene, spanning over 120 kb. This gene has 13 transcripts and consisting of over 38 coding exons which is about 4500 coding nucleotides (8, 14). CPS1D is a rare autosomal recessive disease and can be Lethal hyperammonemia agent. this is a severe type of UCD. Hypothermia, anorexia, vomiting, convulsions, and coma are some of the clinical symptoms of severe CPS1D (15). The most serious complication of CPS1D is Irreversible damage to the central nervous system, which is closely related to the long-term prognosis of neonates (16). Early identification of these signs is vital (17). The one mutation we report herein is located on exon 23 (c.2758G > C; p.Asp920His). This novel homozygous mutations were inherited from our patient's parents, whereas the exact mechanism of dysfunction of human CPS1 caused by this mutation remains unclear. Severe hyperammonemia is the usual presentation of the neonatal onset type and early death often occurs in these cases. Relationship between mutation in the CPS1 and homocysteine has been proved in womenwith RPL and given that heterozygous individuals have no symptoms of homocysteine, further studies are needed to investigate the relationship between CPS1 and abortion (11, 12). Recent study as well as molecular analysis have revealed that mutations in CPS1 gene cannot cause abortion. According to this fact that CPS1 pathogenic variation was found only in 4-6 and considering the healthy persons identifying with 3-4, the aborted fetus in 5-1 cannot be as a result of homozygous mutation in CPS1 gene because its mother doesn't carrying for this mutation. We emphasize the importance of collecting and storing of biological samples. Biochemical assays and gene mutation analysis are helpful in making the diagnosis. Prenatal diagnosis is also available in UCDs. Molecular diagnosis of CPS1D can be hampered by the large size of the CPS1 gene. Genetic analysis is a key element in diagnosing CPS1D and for performing counseling, prenatal diagnosis, and eventually (10), for future procedures of disease-free embryo selection. Despite the fact that prenatal CPS1D diagnosis has been introduced in other populations, there is currently no prenatal diagnosis test in Iran. Today, Development of NGS technologies promising researcher to generate large amounts of sequence data at a lower cost and with less attempt, provided new possibilities for diagnostic pathogenic variant screening (18).

##  Conflict of Interest

The authors declare no conflict of interest.
